# The influence of pulmonary vascular pressures on lung diffusing capacity during incremental exercise in healthy aging

**DOI:** 10.14814/phy2.13565

**Published:** 2018-01-25

**Authors:** Kirsten E. Coffman, Timothy B. Curry, Niki M. Dietz, Steven C. Chase, Alex R. Carlson, Briana L. Ziegler, Bruce D. Johnson

**Affiliations:** ^1^ Mayo Clinic Graduate School of Biomedical Sciences Mayo Clinic Rochester Minnesota; ^2^ Department of Anesthesiology Mayo Clinic Rochester Minnesota; ^3^ Department of Cardiovascular Diseases Mayo Clinic Rochester Minnesota

**Keywords:** Lung diffusing capacity, pulmonary artery pressure, pulmonary capillary recruitment, pulmonary hemodynamics, transpulmonary gradient

## Abstract

Alveolar‐capillary surface area for pulmonary gas exchange falls with aging, causing a reduction in lung diffusing capacity for carbon monoxide (DLCO). However, during exercise additional factors may influence DLCO, including pulmonary blood flow and pulmonary vascular pressures. First, we sought to determine the age‐dependent effect of incremental exercise on pulmonary vascular pressures and DLCO. We also aimed to investigate the dependence of DLCO on pulmonary vascular pressures during exercise via sildenafil administration to reduce pulmonary smooth muscle tone. Nine younger (27 ± 4 years) and nine older (70 ± 3 years) healthy subjects performed seven 5‐min exercise stages at rest, 0 (unloaded), 10, 15, 30, 50, and 70% of peak workload before and after sildenafil. DLCO, cardiac output (*Q*), and pulmonary artery and wedge pressure (mPAP and mPCWP; subset of participants) were collected at each stage. mPAP was higher (*P* = 0.029) and DLCO was lower (*P* = 0.009) throughout exercise in older adults; however, the rate of rise in mPAP and DLCO with increasing *Q* was not different. A reduction in pulmonary smooth muscle tone via sildenafil administration reduced mPAP, mPCWP, and the transpulmonary gradient (TPG = mPAP–mPCWP) in younger and older subjects (*P* < 0.001). DLCO was reduced following the reduction in mPAP and TPG, regardless of age (*P* < 0.001). In conclusion, older adults successfully adapt to age‐dependent alterations in mPAP and DLCO. Furthermore, DLCO is dependent on pulmonary vascular pressures, likely to maintain adequate pulmonary capillary recruitment. The rise in pulmonary artery pressure with aging may be required to combat pulmonary vascular remodeling and maintain lung diffusing capacity, particularly during exercise.

## Introduction

Healthy aging is associated with alterations in both lung diffusing capacity and pulmonary hemodynamics, but the interplay between these variables remains unclear. Lung diffusing capacity, or the ability to transfer gases from the alveoli to blood, is reduced with aging, both at rest and during exercise (Chang et al. 1992; Stam et al. 1994; Guenard and Marthan 1996; Coffman et al. 2017). This is likely in part due to remodeling of the pulmonary alveoli and capillaries (Butler and Kleinerman 1970; Thurlbeck and Angus 1975; Verbeken et al. 1992; Gillooly and Lamb 1993) which acts to reduce surface area available for gas exchange. However, whether age‐dependent changes in pulmonary vascular pressures also alter lung diffusing capacity is not known. A decreased distensibility of the pulmonary vasculature with healthy aging has been described, as determined by both ex vivo studies (Harris et al. 1965; Gozna et al. 1974; Banks et al. 1978; Mackay et al. 1978) and modeling of in vivo systems (Reeves et al. 2005). This increased stiffness of the vessel walls likely contributes to the increases in pulmonary pressures and pulmonary vascular resistance associated with increasing age (Emirgil et al. 1967; Ehrsam et al. 1983; Reeves et al. 2005; Kovacs et al. 2009; Van Empel et al. 2014). Furthermore, it is well established that maximal aerobic capacity (VO_2max_) is reduced in older individuals (Byrne et al. 1996; American Thoracic S, 2003; Fleg et al. 2005), and several studies have demonstrated an association between VO_2max_ and both lung diffusing capacity (Lalande et al. 2012; Coffman et al. 2017) and pulmonary hemodynamics (Fujii et al. 1996; Coffman et al. 2017; Faoro et al. 2017). Therefore, it is feasible that age‐dependent increases in pulmonary vascular pressures may alter the increase in lung surface area available for gas exchange, particularly during exercise.

While both lung diffusing capacity and pulmonary vascular pressures are known to be altered with healthy aging, we are unaware of any study that has measured these variables in concert. The catalyst for investigating the relationship between pulmonary hemodynamics and lung diffusing capacity came from a previous study in our laboratory in which we found that, during incremental exercise, the rate of increase in lung diffusing capacity was remarkably similar in older adults compared to their younger counterparts. Furthermore, in very fit older individuals who might be anticipated to approach the upper limits of pulmonary capillary recruitment, there was no evidence of a limitation in lung diffusing capacity, even during near maximal exercise. Interestingly, however, lung diffusing capacity was observed to be systematically reduced for a given cardiac output (i.e., DLCO/*Q*) throughout incremental exercise in older versus younger individuals (Coffman et al. 2017). In other words, a given cardiac output through the pulmonary vasculature did not yield the same lung surface area for gas exchange at a given exercise level. While this reduced ability to utilize cardiac output to recruit lung surface area for gas exchange is likely partially an outcome of changes to the alveolar‐capillary interface with aging (Butler and Kleinerman 1970; Thurlbeck and Angus 1975; Verbeken et al. 1992; Gillooly and Lamb 1993), it is possible that age‐dependent alterations in pulmonary hemodynamics also play a role.

Thus, there were two primary aims of this study. First, we aimed to investigate the age‐dependent changes in both lung diffusing capacity and pulmonary vascular pressures throughout a slow, incremental exercise bout. Second, we aimed to investigate the dependence of lung diffusing capacity on pulmonary vascular pressures in both younger and older individuals. We hypothesized that older individuals would have elevated pulmonary vascular pressures and reduced lung diffusing capacity for a given cardiac output throughout incremental exercise, but that the rate of rise with exercise would be similar. Additionally, we hypothesized that lung diffusing capacity would be altered by changes in pulmonary hemodynamics via sildenafil administration in older individuals only.

## Methods

### Subjects

Nine younger (4M/5F, 27 ± 4 year) and nine older (5M/4F, 70 ± 3 year) nonsmoking adults with no history of respiratory, cardiovascular, or metabolic disease participated in the study (Table 1; mean ± SD). Participants were recruited from the general population and were moderately active individuals. Each participant gave written informed consent after being provided a detailed description of the study requirements. The experimental procedures were approved by the Mayo Clinic Institutional Review Board and were performed in accordance with the ethical standards of the Declaration of Helsinki.

**Table 1:** Subject demographics for all subjects as well as only those with catheter data

	Younger	Older	*P*‐Value
All subjects			
Subjects, *N* (M/F)	9 (4/5)	9 (5/4)	
Age, y	26.7 ± 3.7	70.0 ± 3.1	<0.001
Height, cm	176 ± 11	170 ± 9	0.223
Weight, kg	71 ± 9.8	71.2 ± 10.6	0.973
BMI, kg/m^2^	22.8 ± 1.9	24.5 ± 2.8	0.151
BSA, m^2^	1.86 ± 0.18	1.83 ± 0.17	0.717
*W*_peak_, watts	228 ± 65	118 ± 37	<0.001
VO_2_ peak, mL/min/kg	39.9 ± 6.4	23.2 ± 3.5	<0.001
FVC, % pred.	106 ± 10	98 ± 9	0.070
FEV_1_, % pred.	102 ± 11	100 ± 12	0.711
FEV_1_/FVC, % pred.	96 ± 5	102 ± 7	0.032
Hemoglobin, g/dL	13.7 ± 1.4	14.0 ± 1.2	0.561
Subset with catheters			
Subjects, *N* (M/F)	4 (2/2)	5 (3/2)	
Age, y	28.6 ± 5.1	69.6 ± 2.7	<0.001
Height, cm	173 ± 5	174 ± 8	0.837
Weight, kg	65.6 ± 8.5	71.2 ± 13.1	0.485
BMI, kg/m^2^	21.9 ± 2	23.4 ± 2.6	0.381
BSA, m^2^	1.77 ± 0.14	1.85 ± 0.21	0.548
*W*_peak_, watts	201 ± 22	125 ± 38	0.010
VO_2peak_, mL/min/kg	38.0 ± 2.7	24.3 ± 3	<0.001
FVC, % pred.	99 ± 6	96 ± 11	0.696
FEV_1_, % pred.	94 ± 4	97 ± 14	0.688
FEV_1_/FVC, % pred.	95 ± 4	100 ± 4	0.088
Hemoglobin, g/dL	13.9 ± 1.3	14.0 ± 0.9	0.842

BMI, body mass index; BSA, body surface area; *W*_peak_, peak work rate; VO_2peak_, peak oxygen consumption; FVC, forced vital capacity; FEV_1_, forced expiratory volume in 1 second.

### Study overview

The experimental procedures were completed during two separate visits to the laboratory. The subjects abstained from caffeine and exercise for 12 h prior to each visit. At visit 1, a blood sample was collected for determination of hemoglobin concentration; participants were excluded if their hemoglobin was below 12.5 g/dL for males or 11.5 g/dL for females. Next, pulmonary function was assessed via full body plethysmography (MedGraphics Elite Series Plethysmograph, Medical Graphics Corporation, St. Paul, MN, USA) according to standard procedures (Miller et al. 2005); subjects were excluded if their pulmonary function (FVC, FEV_1_) was below 80% of predicted (Hankinson et al. 1999). Finally, subjects completed an incremental cycling exercise test to exhaustion for determination of peak work rate (*W*_peak_) and peak oxygen consumption (VO_2peak_). Exercise began at 0, 30, or 60 W and increased by 15 or 30 W every 2 min dependent on self‐reported fitness. *W*_peak_ was calculated as the sum of the final work rate completed plus the fraction of the partially completed work rate before exhaustion. VO_2peak_ was calculated as the 30 sec average immediately preceding the highest VO_2_ value.

At visit 2, insertion of a pulmonary Swan‐Ganz catheter was attempted in all participants. However, the catheterization was only completed in a subset of individuals, who then also underwent insertion of an arterial catheter (4 younger and 5 older, Table 1). Next, subjects performed an identical bout of incremental cycle exercise before and after a pharmacological intervention to reduce pulmonary smooth muscle tone. The exercise protocol included a 5 min rest stage, followed by cycling for 5 min each at 0 (unloaded), 10, 15, 30, 50, and 70% of *W*_peak_. In those individuals with catheters, pulmonary artery and systemic blood pressure were sampled 90 sec into each stage, followed by collection of a wedge pressure (an estimate of pulmonary venous pressure) 2 min into each stage. In those individuals with catheters, arterial and mixed venous blood samples were drawn 3 min into each stage for calculation of cardiac output via direct Fick. In all individuals, estimates of cardiac output (*Q*), as well as lung diffusing capacity for carbon monoxide (DLCO), were measured via a rebreathe technique 4 min into each exercise stage (Hsia et al. 1995; Johnson et al. 2000; Tamhane et al. 2001). Oxygen consumption was also continuously measured throughout the exercise bout and sampled 3 min into each stage for use in the direct Fick calculation for *Q* (BreezeSuite 6.4.1 SP5, Medical Graphics Corporation, St. Paul, MN, USA). After completion of the first exercise bout, 100 mg sildenafil, a pulmonary vasodilator, was administered orally followed by a 1 h rest period in order for sildenafil to take effect. During this rest period, subjects were allowed water and small snacks ad libitum. Then, the exercise bout was repeated.

### Pulmonary artery and wedge pressure via Swan‐Ganz catheter

In a subset of participants (Table 1), a Swan‐Ganz catheter (Swan‐Ganz 123F6, Edwards Lifesciences LLC, Irvine, CA, USA) was inserted under local anesthesia (2% lidocaine) by two experienced anesthesiologists. The catheter was inserted into an antecubital vein and advanced to the pulmonary artery under ultrasound guidance and electrocardiogram monitoring. The proper placement of the catheter was established by observing the pulmonary artery waveform as well as the wedge waveform upon balloon inflation.

As exercise work rate increased, respiratory variations caused cyclic variations in pulmonary artery pressure. Therefore, LabChart (LabChart 8, ADInstruments, Colorado Springs, CO, USA) was used to apply a digital band pass filter to the raw pressure signal in order to remove respiratory frequencies (i.e., the lower filter limit), but preserve cardiac frequencies (i.e., the upper filter limit). The frequency limits were updated with each exercise stage according to the current respiratory rate and heart rate. Importantly, the amplitude of the change in pulmonary artery pressure from diastole to systole was verified to be maintained after filtering. Once the digital filter had been applied, mean pulmonary artery pressure (mPAP) was determined as the 30 sec average starting at 90 sec into each stage. Pulmonary artery systolic (sPAP) and diastolic pressure (dPAP) were also determined as the maximum and minimum value, respectively, within each cardiac cycle over the same 30 sec window.

Next, mean pulmonary capillary wedge pressure (mPCWP) was collected at 2 min into each exercise stage. The balloon at the tip of the catheter was slowly inflated until pulmonary pressure fell to a stable, lower value. The inflation was maintained for ~10 sec, and the pressure over this period was averaged as mPCWP.

### Systemic blood pressure via arterial catheter

An arterial catheter (FA‐04020, Arrow International Inc., Reading, PA, USA) was also placed in the same subset of participants under local anesthesia (2% lidocaine) by an experienced anesthesiologist for continuous measurement of systemic blood pressure. Systemic systolic (sSBP) and diastolic blood pressure (dSBP) were determined as the maximum and minimum value, respectively, within each cardiac cycle. sSBP, sDBP, as well as mean systemic blood pressure (mSBP) were averaged over the same 30 sec window as pulmonary pressures (90 sec into each exercise stage).

### Lung diffusing capacity and cardiac output via rebreathe

Lung diffusing capacity for carbon monoxide (DLCO) and cardiac output (*Q*) were assessed using a rebreathe technique 4 min into each exercise stage in all subjects (Hsia et al. 1995; Johnson et al. 2000; Tamhane et al. 2001). Using this technique, DLCO and *Q* are determined via the rate of disappearance of CO and acetylene (C_2_H_2_), respectively. Briefly, subjects breathed through a two‐way switching valve connected to a pneumotachometer, mass spectrometer (Marquette 1100 Medical Gas Analyser, Perkin‐Elmer, St. Louis, MO, USA) and NO analyzer (Sievers 280i NOA, Sievers, Boulder, CO, USA). The inspiratory port of the switching valve was open to room air or a 6‐L anesthesia bag filled with 0.3% C^18^O, 45 ppm NO, 9% He, 0.6% C_2_H_2_, 35% O_2_, and N_2_ balance. The total volume of gas added to the rebreathe bag was determined as the average tidal volume of the subject during the 30 sec immediately prior to each measurement. Following a normal expiration, subjects were switched into the rebreathe bag and instructed to nearly empty the bag with each breath for 8–10 consecutive breaths at a respiratory rate of at least 32 breaths per min.

### Cardiac output via direct Fick

While all subjects had cardiac output measured via acetylene rebreathing, the subset with catheters also had cardiac output measured via direct Fick and these values were used in all further data analysis. In this subset of individuals, arterial and mixed venous blood samples were collected from the arterial and Swan‐Ganz catheters, respectively, 3 min into each exercise stage. Arterial and mixed venous oxygen content (CaO_2_ and CvO_2_) were determined from the blood samples (ABL90, Radiometer, Brea, CA, USA). Oxygen consumption was also averaged over the 30 sec period that coincided with the blood draws and used in the Fick calculation for cardiac output (*Q*):

$$Q=\;\frac{{\text{VO}}_{2}}{{\text{CaO}}_{2}-{\text{CvO}}_{2}}$$

### Mechanism of sildenafil

100 mg sildenafil was administered orally immediately following the first exercise bout, upon which participants rested for 1 h and were allowed water and small snacks ad libitum. Sildenafil is a PDE5 inhibitor, which increases levels of cGMP within smooth muscle cells thus eliciting pulmonary smooth muscle cell relaxation.

### Statistical analyses

**Figure 1:**
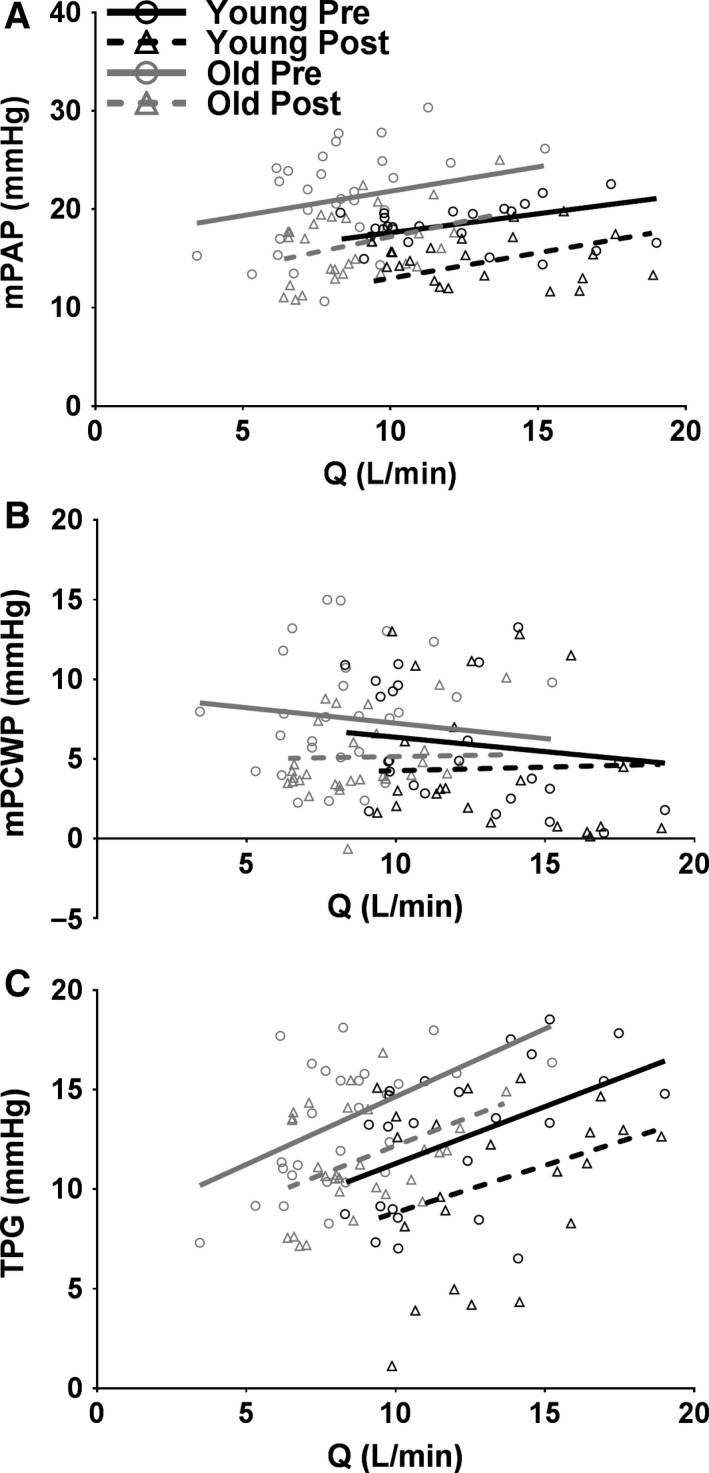
Individual values of mean pulmonary artery pressure (mPAP); (A), mean pulmonary wedge pressure (mPCWP); (B), and transpulmonary gradient (TPG); (C) as a function of cardiac output (*Q*; direct Fick) in those subjects with catheters (*N* = 4 younger and 5 older) during incremental exercise in younger (black) and older (gray) healthy adults presildenafil (circles and solid line) and postsildenafil (triangles and dotted line). The data were fit using a linear mixed effects model. mPAP is significantly greater in older adults (A; *P* = 0.029). mPAP, mPCWP, and TPG are significantly reduced following sildenafil administration (all *P* < 0.001).

**Figure 2:**
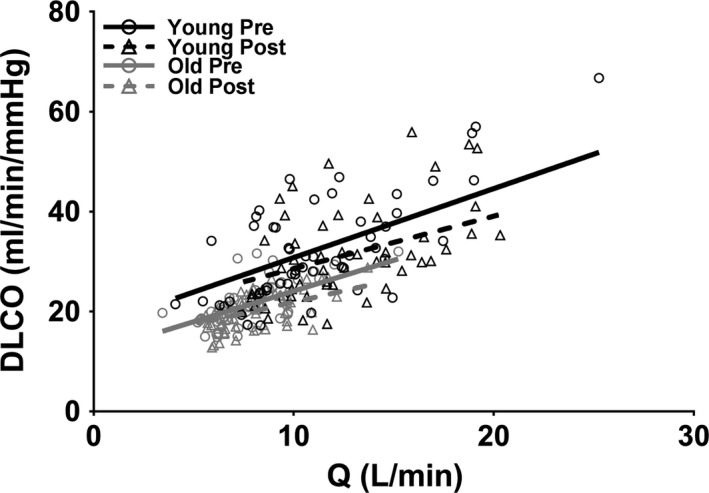
Individual values of lung diffusing capacity for carbon monoxide (DLCO) as a function of cardiac output (*Q*) in all subjects (*N* = 9 younger and 9 older) during incremental exercise in younger (black) and older (gray) healthy adults presildenafil (circles and solid line) and postsildenafil (triangles and dotted line). Measures of *Q* are a combination of direct Fick in those subjects with catheters and acetylene rebreathing in those subjects without catheters. The data were fit using a linear mixed effects model. DLCO is significantly reduced following sildenafil administration (*P* < 0.001).

**Figure 3:**
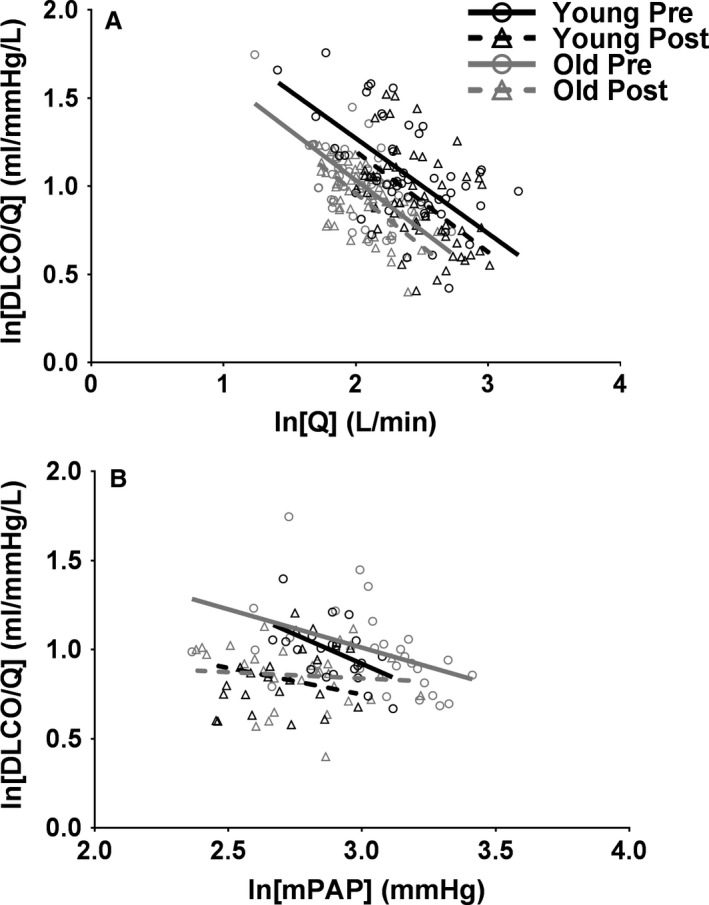
Lung diffusing capacity for a given blood flow through the pulmonary vasculature (DLCO/*Q*) as a function of cardiac output (*Q*); (A) or mean pulmonary artery pressure (mPAP); (B) during incremental exercise in younger (black) and older (gray) healthy adults presildenafil (circles and solid line) and postsildenafil (triangles and dotted line). Figure A, which does not incorporate mPAP, consists of all subjects (*N* = 9 younger and 9 older) and measures of *Q* are a combination of direct Fick in those with catheters and acetylene rebreathing in those without catheters. Figure B, which incorporates mPAP, consists of only those subjects with catheters (*N* = 4 younger and 5 older) and measures of Q are direct Fick. Data were linearized to simplify statistical analyses and the data were fit using a linear mixed effects model.

Independent samples *t*‐test was used to compare younger versus older individuals (Table 1). Figures 1, 2, 3 were analyzed using a linear mixed effects model to determine both the effect of healthy aging and sildenafil administration on (1) the response to incremental exercise, that is, the slope of the given relationship, if there was a significant relationship, and (2) the mean value of the independent variable, that is, the offset or vertical shift in the datasets. In short, using this model the linear fit through the data is obtained by taking into account repeated measures (i.e., multiple exercise stages for each subject) and effectively averaging the individual subject responses. Thus, the line of fit may appear different than it would if a line were fit to all data points without accounting for individual subject relationships. All data are expressed as mean ± SD and all statistical analyses were performed in Matlab (version R2016a, Natick, MA) with statistical significance set at *P* < 0.05.

## Results

### Subjects

Subject characteristics and pulmonary function are shown in Table 1, including data for all subjects that participated in the study (“All Subjects”) as well as only the subset of individuals who underwent pulmonary and arterial catheter insertion (“Subset with Catheters”). In both cases, the younger and older individuals were well‐matched for height, weight, BMI, and BSA. *W*_peak_ and VO_2peak_ were lower in older versus younger subjects (*P* = 0.010 and *P* < 0.001, respectively, Table 1). Additionally, percent‐predicted FVC and FEV_1_ were not different between younger and older individuals; however, percent‐predicted FEV_1_/FVC was slightly lower in the younger versus older individuals in the complete dataset (96 ± 5 vs. 102 ± 7%, *P* = 0.032).

### Pulmonary vascular response to incremental exercise

#### Pulmonary vascular pressures

Figure 1A shows mPAP as a function of *Q*. In all conditions, mPAP increased with increasing *Q* (all *P* ≤ 0.010). While older individuals did not demonstrate a differential pressure response (i.e., slope) to increasing flow, older individuals had a greater overall mPAP by 4.3 mmHg (*P* = 0.029). A reduction in pulmonary smooth muscle tone via sildenafil administration did not alter the pressure response to increasing flow through the pulmonary vasculature; however, baseline mPAP was significantly reduced in all subjects by 4.6 mmHg (*P* < 0.001). These data suggest that older individuals have higher mean pulmonary artery pressures throughout exercise, and that, as would be expected, a reduction in pulmonary smooth muscle tone significantly reduced pulmonary artery pressure in both younger and older individuals.

Figure 1B shows mPCWP as a function of *Q*. In all conditions there was no significant relationship, meaning that mPCWP did not increase or decrease throughout incremental exercise in younger or older individuals. Furthermore, there was no difference in overall values of mPCWP in younger versus older individuals. However, sildenafil administration significantly reduced baseline mPCWP in younger and older individuals by 2 mmHg (*P* < 0.001). These data suggest that younger and older individuals have similar pulmonary venous pressures and that a reduction in pulmonary smooth muscle tone does in fact reduce pulmonary venous pressure, regardless of age.

Figure 1C shows the transpulmonary gradient (TPG = mPAP–mPCWP) as a function of *Q*. TPG is a measure of the pressure difference across the pulmonary vasculature and can be considered the effective driving pressure through the pulmonary capillary network. In all conditions, TPG increased as *Q* increased (*P* ≤ 0.016). Furthermore, baseline TPG was significantly reduced following sildenafil administration by 2.5 mmHg (*P* < 0.001). These data suggest that a reduction in pulmonary smooth muscle tone significantly reduced the driving pressure through the pulmonary capillaries in both younger and older adults.

Taken together, Figure 1 suggest that older individuals have a higher baseline pulmonary artery, and not pulmonary venous, pressure throughout incremental exercise. However, the pressure response to exercise is remarkably similar in older versus younger individuals. Furthermore, a reduction in pulmonary smooth muscle tone via sildenafil administration significantly reduced both pulmonary artery and venous pressures, regardless of age. Importantly, a reduction in pulmonary smooth muscle tone significantly reduced the transpulmonary gradient, or driving pressure across the pulmonary capillary network, regardless of age.

#### Lung diffusing capacity

The effect of healthy aging and sildenafil administration on DLCO as a function of *Q* is shown in Figure 2. In all conditions, DLCO increased with increasing *Q* (all *P* < 0.001) and older individuals demonstrated a baseline reduction in DLCO of 6.7 ml/min/mmHg throughout exercise (*P* = 0.009). Nevertheless, the DLCO *response* to exercise (i.e., slope) was not different in younger versus older individuals. Interestingly, sildenafil administration *hindered* the DLCO response to exercise, decreasing the DLCO‐*Q* slope by 0.33 mL/mmHg/L (*P* < 0.001). This reduced ability to increase DLCO for a given increase in *Q* led to an overall reduction in DLCO of ~2 mL/min/mmHg at *Q* = 10 L/min and ~4 mL/min/mmHg at *Q* = 15 L/min (both *P* < 0.001). These data confirm that older individuals demonstrate a decreased ability to achieve lung surface area for gas exchange given a certain blood flow through the pulmonary vasculature. Furthermore, a reduction in pulmonary smooth muscle tone, leading to a fall in the transpulmonary gradient or driving force across the pulmonary capillary network, actually impaired DLCO throughout incremental exercise, regardless of age.

#### Relationship between *Q*, mPAP, and DLCO

Another aim of this study was to investigate the dependence of lung diffusing capacity on pulmonary hemodynamics during incremental exercise. In all conditions, DLCO/*Q* significantly decreased with increasing *Q* (Fig. 3A, all *P* < 0.001), suggesting that cardiac output increased out of proportion with lung diffusing capacity during incremental exercise. Additionally, baseline DLCO/*Q* was significantly lower throughout exercise in older versus younger individuals by 1.28 mL/mmHg/L (Fig. 3A, *P* = 0.007). This suggests that, even when normalized to a certain blood flow through the pulmonary vascular network, DLCO is systematically lower in older individuals. Following sildenafil administration, DLCO/*Q* was overall reduced in both younger and older adults by 1.09 mL/mmHg/L (Fig. 3A, *P* < 0.001). This suggests that a reduction in pulmonary smooth muscle tone hinders the ability of the pulmonary vascular system to achieve lung surface area for gas exchange, even when normalized to a certain blood flow. Nevertheless, the DLCO/*Q* response to exercise is remarkably similar in younger versus older individuals, as is the role of pulmonary smooth muscle tone in maintaining DLCO.

Figure 3B shows DLCO/*Q* as a function of mPAP. While DLCO/*Q* was significantly correlated with mPAP before sildenafil administration in younger and older individuals (*P* ≤ 0.003), there was no longer a significant correlation between DLCO/*Q* and mPAP following a reduction in pulmonary smooth muscle tone in either age cohort. Additionally, baseline DLCO/*Q* was reduced as a function of mPAP following sildenafil administration by 1.25 mL/mmHg/L (Fig. 3B, *P* < 0.001). These data suggest that during incremental exercise, regardless of age, the ability of a given blood flow to recruit lung surface area for gas exchange in related to the mean pressure entering the vascular system. However, when pulmonary smooth muscle tone is reduced, the link between DLCO/*Q* and mPAP is disturbed. Nevertheless, the DLCO/*Q* response to exercise remains unaltered by healthy aging, and the role of pulmonary smooth muscle tone is not different in older versus younger adults.

## Discussion

### Major findings

In this study, we characterized the effect of healthy aging on the response of mPAP and DLCO to incremental exercise. Furthermore, we investigated the role of pulmonary hemodynamics on DLCO. The main findings were that: (1) healthy aging was associated with an overall increase in mPAP and an overall decrease in DLCO throughout exercise; however, the rate of rise of mPAP and DLCO during exercise was remarkably similar in older versus younger adults, (2) a reduction in pulmonary smooth muscle tone via sildenafil administration significantly reduced mPAP and thus TPG, in both younger and older adults, and (3) the reduction in mPAP and TPG with sildenafil administration elicited a reduction in DLCO in both younger and older adults. All in all these data suggest that, although older individuals experience systematic changes in pulmonary pressures and DLCO, the ability to respond appropriately to incremental exercise is preserved during healthy aging. Furthermore, these data demonstrate the importance of a sufficient driving pressure across the pulmonary capillary network in order to maintain sufficient pulmonary capillary recruitment, and thus adequate lung surface area for gas exchange, during incremental exercise regardless of age.

### Pulmonary vascular response to aging and incremental exercise

#### Lung diffusing capacity

In agreement with previous studies, DLCO rose throughout exercise in both younger and older individuals (Johnson et al. 1960; Hsia et al. 1995; Tamhane et al. 2001). More interestingly, the rate of rise of DLCO was not different in younger versus older individuals (Fig. 2). This confirms a previous study from our laboratory that found that both fitness and age do not alter the DLCO‐*Q* slope throughout incremental exercise (Coffman et al. 2017). We also observed a systematic reduction in DLCO throughout exercise in older individuals; this is also in agreement with the previous study from our laboratory (Coffman et al. 2017). Theoretically, this reduction in DLCO for a given blood flow through the system could be a result of increased alveoli diameter (Butler and Kleinerman 1970; Thurlbeck and Angus 1975; Verbeken et al. 1992; Gillooly and Lamb 1993) and/or reduced pulmonary capillary density with aging (Butler and Kleinerman 1970), both of which may reduce lung surface area for gas exchange. Nevertheless, even given the reduction in DLCO for a given *Q*, the rate at which DLCO increases in unchanged.

#### Mean pulmonary artery pressure

Also in agreement with previous studies, mPAP rose during exercise in both younger and older individuals (Emirgil et al. 1967; Ehrsam et al. 1983; Reeves et al. 2005; Kovacs et al. 2009; Van Empel et al. 2014). However, we did not observe a significant effect of age on the mPAP‐*Q* slope during incremental exercise (Fig. 1A); this finding is in contrast with previous studies that have shown a greater mPAP‐*Q* slope from rest to exercise in older individuals (Emirgil et al. 1967; Reeves et al. 2005). We also found that healthy aging was associated with a systematic increase in mPAP throughout incremental exercise. This confirms previous studies that have demonstrated a higher mPAP during exercise with increasing age (Emirgil et al. 1967; Ehrsam et al. 1983; Reeves et al. 2005; Kovacs et al. 2009; Van Empel et al. 2014). Nevertheless, the unique combination of DLCO and mPAP measurements in this study yielded novel insights into pulmonary capillary distension, recruitment, and the importance of pulmonary vascular pressures in the maintenance of lung surface area for gas exchange, discussed next.

#### Pulmonary capillary recruitment versus distension during exercise

DLCO/*Q* fell throughout exercise in both younger and older adults (Fig. 3A). These data demonstrate that, during incremental exercise, *Q* increased out of proportion with DLCO. In other words, a given increase in flow through the pulmonary vasculature did not cause a proportional increase in lung diffusing capacity. Theoretically, a fall in DLCO/*Q* might suggest that, during exercise, the pulmonary vasculature accepts increases in blood flow at least partially via distension of already perfused capillaries; in contrast, exclusive recruitment of additional pulmonary capillaries would be expected to increase lung surface area for gas exchange in equal proportions with the increase in available cardiac output. This is in agreement with previous hypotheses that an increase in blood flow through the pulmonary vasculature during exercise is at least partially due to pulmonary capillary distension in both older and younger adults (Glenet et al. 2007; Lalande et al. 2012; Pavelescu et al. 2013; Coffman et al. 2017). Nevertheless, additional data would be needed to make any conclusions regarding the relative extent of pulmonary capillary distension versus recruitment during exercise.

### Effect of pulmonary smooth muscle tone on pulmonary vascular pressures

In this study, 100 mg sildenafil, a PDE5 inhibitor, was utilized to reduce pulmonary smooth muscle tone. Following sildenafil administration, both mPAP and mPCWP were significantly reduced in all subjects. However, the fall in mPAP was greater, and as such TPG, or the driving pressure across the pulmonary capillary network, was also significantly reduced in all subjects. The fall in TPG is likely an important factor in the ability to distend and recruit pulmonary capillaries during incremental exercise and will be discussed in more detail later (see “Discussion – Importance of TPG in pulmonary capillary recruitment”). Nevertheless, the fall in mPAP and TPG was not significantly different in older versus younger adults, suggesting that pulmonary smooth muscle tone does play a role in pulmonary pressures but is not altered by healthy aging.

### Influence of mPAP on DLCO and DLCO/*Q*

While DLCO/*Q* can elucidate the relative level of distension versus recruitment as exercise progresses, DLCO/*Q* can also speak to the level of lung diffusing capacity that is achieved for a given cardiac output, or blood flow through the pulmonary vasculature. Presently, aging and sildenafil administration were both found to alter DLCO and DLCO/*Q* systematically throughout exercise. These observations elucidate the importance of mPAP on DLCO.

Exercise‐dependent increases in DLCO are generally thought to be primarily an outcome of increases in cardiac output, which in turn increases blood volume within the pulmonary vasculature and subsequently lung surface area for gas exchange. However, this study suggests that mPAP and TPG also play a vital role in maintaining adequate DLCO, particularly as exercise intensity increases. Specifically, following a reduction in mPAP and therefore TPG, there was a significant reduction in DLCO for a given *Q* in both younger and older adults, and this reduction became more severe at higher exercise intensities (Fig. 2). Thus, these data suggest that a baseline level of pulmonary smooth muscle tone is required to maintain DLCO, particularly at higher levels of exercise. A potential mechanism for the dependence of DLCO on pulmonary pressures is discussed later (see “Importance of TPG in pulmonary capillary recruitment”).

The observation that healthy aging is associated with a baseline reduction in DLCO/*Q* as a function of mPAP during exercise is another novel aspect of this study. That DLCO/*Q* is reduced for a given mPAP suggests that a given blood pressure entering the pulmonary circulation yields a reduced level of alveolar‐capillary surface area in older individuals. In other words, in older individuals a greater mPAP is required to achieve the same DLCO as in a younger individual. This finding suggests that a greater mPAP may be required to maintain DLCO, perhaps due to increases in pulmonary vascular remodeling, with healthy aging. In other words, it is feasible that, in order to overcome pulmonary vascular remodeling, mPAP must increase in older individuals in order to maintain TPG and thus DLCO.

### Importance of TPG in pulmonary capillary recruitment

The mechanism responsible for the dependence of DLCO and DLCO/*Q* on mPAP is of interest. Theoretically, it is possible that a certain transpulmonary gradient (TPG), or driving pressure across the pulmonary capillary network, is required in order to sufficiently recruit additional pulmonary capillaries during exercise, particularly in upper regions of the lung. For example, a reduction in TPG might cause pulmonary capillary derecruitment in upper regions of the lung, as the driving pressure for blood would be insufficient. Thus, blood would effectively pool in the lower regions of the lung and recruitment of additional lung surface area would be reduced. While only speculative, this derecruitment of pulmonary capillaries may explain why the same cardiac output through the pulmonary vasculature results in a significantly lower DLCO following sildenafil administration. In other words, a given cardiac output through the pulmonary vasculature might be less effective at recruiting lung surface area for gas exchange. All in all, the dependence of DLCO on mPAP and TPG suggests that a fall in pulmonary pressures hinders DLCO, independent of age.

The reduction in DLCO following a reduction in mPAP is in contrast to previous studies demonstrating that, in clinical conditions where pulmonary vascular pressures are increased, sildenafil actually improves DLCO (Guazzi et al. 2004a,b; Bussotti et al. 2008; Vitulo et al. 2017). Theoretically, this may be evidence of a ‘sweet spot’ for mPAP. In this study, a certain transpulmonary gradient, driven by mPAP, was required to maintain DLCO during incremental exercise. However, whether this gradient can become too large, such that DLCO is actually reduced, may be the case in diseases where mPAP is increased above normal. In this way, adequate mPAP may be required to sufficiently distribute pulmonary capillary blood through the vascular network, but too high of mPAP may hinder cardiac output through the pulmonary vasculature. Furthermore, it is feasible that this ‘sweet spot’ for mPAP may be greater in older individuals in order to combat increases in pulmonary vascular remodeling. Thus, there may be a fine balance between adequate and deleterious pulmonary artery pressure in regard to lung diffusing capacity.

### Technical considerations

The gas mixture used to measure lung diffusing capacity included 45 ppm NO, a potent vasodilator. It has been shown that continuous inhalation of NO (40 ppm) for 5 min yields changes in regional pulmonary blood flow (Asadi et al. 2015). Thus, it is possible that the rebreathe maneuver used to measure DLCO may alter ventilation‐perfusion matching. However, the DLCO measurement used presently incorporates only a single bolus of 40 ppm NO which is rebreathed for ~15 sec. Therefore, the total NO delivered to the participant is substantially less than that found to alter regional pulmonary blood flow (Asadi et al. 2015). Additionally, while the normoxic condition was found to have an effect in the study by Asadi and colleagues, the effects on ventilation‐perfusion matching appeared much more pronounced in the hypoxic condition (when vasoconstriction would be anticipated to be greater prior to NO inhalation). Thus, in this study the effects of a single NO bolus on ventilation‐perfusion matching in normoxic conditions are likely minimal.

## Conclusions

In conclusion, we found that healthy older individuals had a greater pulmonary artery pressure and lower lung diffusing capacity for a given cardiac output throughout exercise versus younger individuals. However, the rate of rise of DLCO and mPAP with exercise was remarkably similar between younger and older adults, suggesting that older individuals successfully adapt to age‐dependent alterations in the pulmonary vasculature. Interestingly, we found that DLCO is dependent on mPAP in both younger and older adults, such that a decrease in mPAP elicits a reduction in DLCO. These data suggest that a sufficient transpulmonary gradient is required to maintain adequate pulmonary capillary recruitment during exercise. Furthermore, we hypothesize that the age‐dependent increase in mPAP may occur in order to combat pulmonary vascular remodeling, such that the driving pressure for pulmonary capillary recruitment is maintained, and therefore DLCO not hindered, in older individuals.

## References

[phy213565-bib-0001] American Thoracic S and American College of Chest P . 2003 Ats/Accp statement on cardiopulmonary exercise testing. Am. J. Respir. Crit. Care Med. 167:211–277. 12524257 10.1164/rccm.167.2.211

[phy213565-bib-0002] Asadi, A. K. , R. C. Sa , N. H. Kim , R. J. Theilmann , S. R. Hopkins , R. B. Buxton , et al. 2015 Inhaled nitric oxide alters the distribution of blood flow in the healthy human lung, suggesting active hypoxic pulmonary vasoconstriction in normoxia. J. Appl. Physiol. (1985) 118:331–343. 25429099 10.1152/japplphysiol.01354.2013 PMC4312852

[phy213565-bib-0003] Banks, J. , F. V. Booth , E. H. Mackay , B. Rajagopalan , and G. D. Lee . 1978 The physical properties of human pulmonary arteries and veins. Clin. Sci. Mol. Med. 55:477–484. 720001 10.1042/cs0550477

[phy213565-bib-0004] Bussotti, M. , P. Montorsi , M. Amato , A. Magini , D. Baldassarre , F. Tantardini , et al. 2008 Sildenafil improves the alveolar‐capillary function in heart failure patients. Int. J. Cardiol. 126:68–72. 17490765 10.1016/j.ijcard.2007.03.118

[phy213565-bib-0005] Butler, C. 2nd , and J. Kleinerman . 1970 Capillary density: alveolar diameter, a morphometric approach to ventilation and perfusion. Am. Rev. Respir. Dis. 102:886–894. 5486221 10.1164/arrd.1970.102.6.886

[phy213565-bib-0006] Byrne, E. A. , J. L. Fleg , P. V. Vaitkevicius , J. Wright , and S. W. Porges . 1996 Role of aerobic capacity and body mass index in the age‐associated decline in heart rate variability. J. Appl. Physiol. (1985) 81:743–750. 8872642 10.1152/jappl.1996.81.2.743

[phy213565-bib-0007] Chang, S. C. , H. I. Chang , S. Y. Liu , G. M. Shiao , and R. P. Perng . 1992 Effects of body position and age on membrane diffusing capacity and pulmonary capillary blood volume. Chest 102:139–142. 1623741 10.1378/chest.102.1.139

[phy213565-bib-0008] Coffman, K. E. , A. R. Carlson , A. D. Miller , B. D. Johnson , and B. J. Taylor . 2017 The effect of aging and cardiorespiratory fitness on the lung diffusing capacity response to exercise in healthy humans. J. Appl. Physiol. (1985) 122:1425–1434. 28336536 10.1152/japplphysiol.00694.2016 PMC5494432

[phy213565-bib-0009] Ehrsam, R. E. , A. Perruchoud , M. Oberholzer , F. Burkart , and H. Herzog . 1983 Influence of age on pulmonary haemodynamics at rest and during supine exercise. Clin. Sci. (Lond.) 65:653–660. 6627850 10.1042/cs0650653

[phy213565-bib-0010] Emirgil, C. , B. J. Sobol , S. Campodonico , W. H. Herbert , R. Mechkati . 1967 Pulmonary circulation in the aged. J. Appl. Physiol. 23:631–640. 6061377 10.1152/jappl.1967.23.5.631

[phy213565-bib-0011] Faoro, V. , G. Deboeck , M. Vicenzi , A. F. Gaston , B. Simaga , G. Doucende , et al. 2017 Pulmonary vascular function and aerobic exercise capacity at moderate altitude. Med. Sci. Sports Exerc. 49:2131–2138. 28915226 10.1249/MSS.0000000000001320

[phy213565-bib-0012] Fleg, J. L. , C. H. Morrell , A. G. Bos , L. J. Brant , L. A. Talbot , J. G. Wright , et al. 2005 Accelerated longitudinal decline of aerobic capacity in healthy older adults. Circulation 112:674–682. 16043637 10.1161/CIRCULATIONAHA.105.545459

[phy213565-bib-0013] Fujii, T. , N. Kurihara , S. Fujimoto , K. Hirata , and J. Yoshikawa . 1996 Role of pulmonary vascular disorder in determining exercise capacity in patients with severe chronic obstructive pulmonary disease. Clin. Physiol. 16:521–533. 8889315 10.1111/j.1475-097x.1996.tb01017.x

[phy213565-bib-0014] Gillooly, M. , and D. Lamb . 1993 Airspace size in lungs of lifelong non‐smokers: effect of age and sex. Thorax 48:39–43. 8434351 10.1136/thx.48.1.39 PMC464237

[phy213565-bib-0015] Glenet, S. N. , C. De Bisschop , F. Vargas , and H. J. Guenard . 2007 Deciphering the nitric oxide to carbon monoxide lung transfer ratio: physiological implications. J. Physiol. 582:767–775. 17495039 10.1113/jphysiol.2007.133405 PMC2075329

[phy213565-bib-0016] Gozna, E. R. , A. E. Marble , A. Shaw , and J. G. Holland . 1974 Age‐related changes in the mechanics of the aorta and pulmonary artery of man. J. Appl. Physiol. 36:407–411. 4820321 10.1152/jappl.1974.36.4.407

[phy213565-bib-0017] Guazzi, M. , G. Tumminello , Marco F. Di , C. Fiorentini , C. Fiorentini , and M. D. Guazzi . 2004a The effects of phosphodiesterase‐5 inhibition with sildenafil on pulmonary hemodynamics and diffusion capacity, exercise ventilatory efficiency, and oxygen uptake kinetics in chronic heart failure. J. Am. Coll. Cardiol. 44:2339–2348. 15607396 10.1016/j.jacc.2004.09.041

[phy213565-bib-0018] Guazzi, M. , G. Tumminello , F. Di Marco , and M. D. Guazzi . 2004b Influences of sildenafil on lung function and hemodynamics in patients with chronic heart failure. Clin. Pharmacol. Ther. 76:371–378. 15470337 10.1016/j.clpt.2004.06.003

[phy213565-bib-0019] Guenard, H. , and R. Marthan . 1996 Pulmonary gas exchange in elderly subjects. Eur. Respir. J. 9:2573–2577. 8980971 10.1183/09031936.96.09122573

[phy213565-bib-0020] Hankinson, J. L , J. R. Odencrantz , and K. B. Fedan . 1999 Spirometric reference values from a sample of the general U.S. population. Am. J. Respir. Crit. Care Med. 159:179–187. 9872837 10.1164/ajrccm.159.1.9712108

[phy213565-bib-0021] Harris, P. , D. Heath , and A. Apostolopoulos . 1965 Extensibility of the human pulmonary trunk. Br. Heart. J. 27:651–659. 5829747 10.1136/hrt.27.5.651 PMC469755

[phy213565-bib-0023] Hsia, C. C. , D. G. Mcbrayer , and M. Ramanathan . 1995 Reference values of pulmonary diffusing capacity during exercise by a rebreathing technique. Am. J. Respir. Crit. Care Med. 152:658–665. 7633723 10.1164/ajrccm.152.2.7633723

[phy213565-bib-0025] Johnson, R. L. Jr , W. S. Spicer , J. M. Bishop , and R. E. Forster . 1960 Pulmonary capillary blood volume, flow and diffusing capacity during exercise. J. Appl. Physiol. 15:893–902. 13790336 10.1152/jappl.1960.15.5.893

[phy213565-bib-0026] Johnson, B. D. , K. C. Beck , D. N. Proctor , J. Miller , N. M. Dietz , and M. J. Joyner . 2000 Cardiac output during exercise by the open circuit acetylene washin method: comparison with direct fick. J. Appl. Physiol. 88:1650–1658. 10797126 10.1152/jappl.2000.88.5.1650

[phy213565-bib-0027] Kovacs, G. , A. Berghold , S. Scheidl , and H. Olschewski . 2009 Pulmonary arterial pressure during rest and exercise in healthy subjects: a systematic review. Eur. Respir. J. 34:888–894. 19324955 10.1183/09031936.00145608

[phy213565-bib-0028] Lalande, S. , P. Yerly , V. Faoro , and R. Naeije . 2012 Pulmonary vascular distensibility predicts aerobic capacity in healthy individuals. J. Physiol. 590:4279–4288. 22733662 10.1113/jphysiol.2012.234310 PMC3473285

[phy213565-bib-0029] Mackay, E. H. , J. Banks , B. Sykes , and G. Lee . 1978 Structural basis for the changing physical properties of human pulmonary vessels with age. Thorax 33:335–344. 684670 10.1136/thx.33.3.335 PMC470893

[phy213565-bib-0030] Miller, M. R. , J. Hankinson , V. Brusasco , F. Burgos , R. Casaburi , A. Coates , et al. 2005 Standardisation of spirometry. Eur. Respir. J. 26:319–338. 16055882 10.1183/09031936.05.00034805

[phy213565-bib-0031] Pavelescu, A. , V. Faoro , H. Guenard , C. De Bisschop , J. B. Martinot , C. Melot , et al. 2013 Pulmonary vascular reserve and exercise capacity at sea level and at high altitude. High Alt. Med. Biol. 14:19–26. 23537256 10.1089/ham.2012.1073

[phy213565-bib-0033] Reeves, J. T. , J. H. Linehan , and K. R. Stenmark . 2005 Distensibility of the normal human lung circulation during exercise. Am. J. Physiol. Lung Cell. Mol. Physiol. 288:L419–L425. 15695542 10.1152/ajplung.00162.2004

[phy213565-bib-0034] Stam, H. , V. Hrachovina , T. Stijnen , and A. Versprille . 1994 Diffusing capacity dependent on lung volume and age in normal subjects. J. Appl. Physiol. 76:2356–2363. 7928858 10.1152/jappl.1994.76.6.2356

[phy213565-bib-0035] Tamhane, R. M. , R. L. Jr Johnson , and C. C. Hsia . 2001 Pulmonary membrane diffusing capacity and capillary blood volume measured during exercise from nitric oxide uptake. Chest 120:1850–1856. 11742912 10.1378/chest.120.6.1850

[phy213565-bib-0036] Thurlbeck, W. M. , and G. E. Angus . 1975 Growth and aging of the normal human lung. Chest 67:3s–6s. 1112103 10.1378/chest.67.2_supplement.3s

[phy213565-bib-0037] Van Empel, V. P. , D. M. Kaye , and B. A. Borlaug . 2014 Effects of healthy aging on the cardiopulmonary hemodynamic response to exercise. Am. J. Cardiol. 114:131–135. 24852914 10.1016/j.amjcard.2014.04.011

[phy213565-bib-0038] Verbeken, E. K. , M. Cauberghs , I. Mertens , J. Clement , J. M. Lauweryns , and K. P. Van De Woestijne . 1992 The senile lung. comparison with normal and emphysematous lungs. 1. Structural aspects. Chest 101:793–799. 1541148 10.1378/chest.101.3.793

[phy213565-bib-0039] Vitulo, P. , A. Stanziola , M. Confalonieri , D. Libertucci , T. Oggionni , P. Rottoli , et al. 2017 Sildenafil in severe pulmonary hypertension associated with chronic obstructive pulmonary disease: a randomized controlled multicenter clinical Trial. J. Heart Lung Transplant. 36:166–174. 27329400 10.1016/j.healun.2016.04.010

